# Delayed onset hypokinetic-rigid syndrome due to hypoxic-ischemic damage of the striatum

**DOI:** 10.1007/s13760-016-0712-4

**Published:** 2016-11-18

**Authors:** Walid Moudrous, Menno Sluzewski, Jan-Thies van Asseldonk

**Affiliations:** 1 Department of Neurology, ETZ, location St. Elisabeth Hospital, PO Box 90151, Tilburg, 5000 LC The Netherlands; 2 Department of Radiology, ETZ location St. Elisabeth Hospital, PO Box 90151, Tilburg, 5000 LC The Netherlands

**Keywords:** Hypoxia, Delayed post anoxic encephalopathy, MR pattern, Hypokinetic-rigid syndrome

## Introduction

A 43 years old woman was consulted in the psychiatric ward for acute signs of catatonia. She was scheduled for electroconvulsive therapy. Her medical history showed a borderline personality disorder, depression and back surgery. Six  weeks preceding our consultation, she had a cardiac arrest following an auto-intoxication with benzodiazepines. She was resuscitated 30 min, followed by cooling for 24 h. Four days later she was discharged from the hospital without any neurological sequela. After 6 weeks she was re-admitted for severe rigidity and hypokinesia with a subacute onset. At examination she was comprehensive but mutistic. She was able to communicate by pointing at a letter chart with her eyes. Her limbs were symmetrical hypokinetic and rigid with cogwheel phenomenon. Due to the hypokinetic-rigid syndrome she was bedbound.

**Fig. 1:**
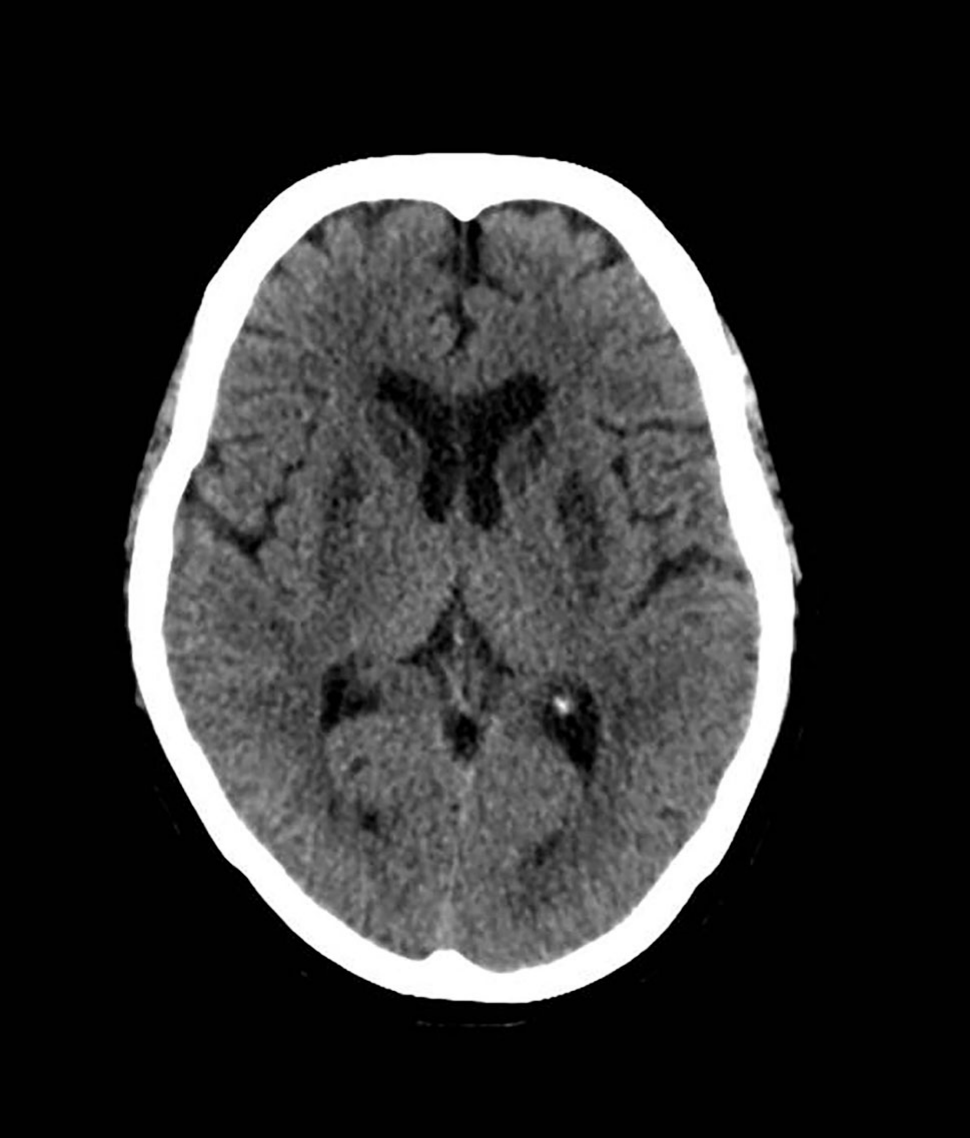
A non-contrast head CT, 6 weeks after cardiac resuscitation, showing symmetrical hypodensity of caudate nuclei and putamen

**Fig. 2:**
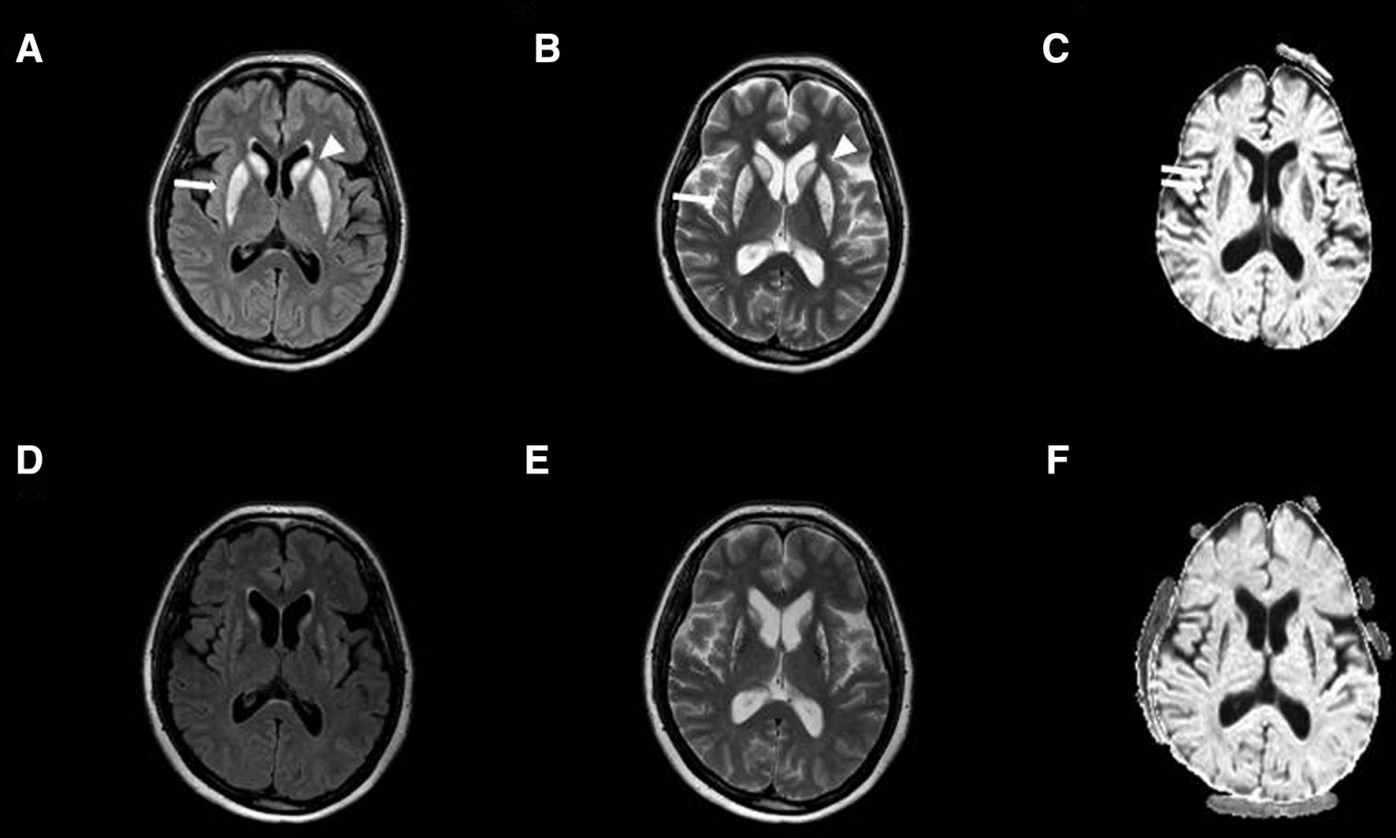
Axial brain imaging in the acute phase, showing increased signal intensity in T2 (**a**) and FLAIR (**b**) imaging of the nuclei caudatus (*arrow head*) and putamen (*arrow*) with sparing of the globus pallidus. DWI/ADC imaging in **c** with diffuse restriction in putamen matching ischemic infarction (*double arrows*). Axial brain imaging after 2 years follow-up, showing gliosis in T2/FLAIR and DWI/ADC (**d**–**f**) of the nuclei caudatus and putamen

A non-contrast head CT was performed, which revealed a symmetric hypodense signal in the caudate nucleus and the putamen (Fig. 1). An additional MRI bilaterally showed a hyperintense signal in the caudate nuclei and the putamen (Fig. 2a–c). Laboratory examination showed no abnormalities. The hypokinetic-rigid syndrome improved on levodopa therapy. Three weeks later at discharge she received 250 mg levodopa 4 times daily and 125 mg slow release at the evening. Although some rigidity and hypokinesia remained, she regained independent living. Additional follow-up showed no further deterioration, and levodopa was halved in dosage. Two years later a follow-up MRI showed that the ischemic structures had become atrophic (Fig. 2d–f).

## Discussion

We present a case with a severe hypokinetic-rigid syndrome due to delayed hypoxic-ischemic brain injury after benzodiazepines intoxication and cardiac resuscitation. In adulthood, the combination of a lucid interval and selective involvement of the basal ganglia is a rare finding following cerebral hypoxia [1–5]. The basal ganglia are highly at risk in anoxic injury because their perfusion is received from a vascular boundary zone. Furthermore, the basal ganglia have a high metabolic demand [2–4]. The pathophysiology of the lucid interval after the hypoxic event is not well understood, but delayed demyelination following acute necrosis has been proposed. Selective basal ganglia involvement is mostly seen in pediatric patients suffering neonatal asphyxia. In adults, it is seldom reported and mainly caused by monoxide poisoning or substance abuse like heroin and benzodiazepines [2]. MR imaging can confirm the signs of hypoxic-ischemic brain injury which is mainly located in the periventricular subcortical white matter, with infratentorial sparing [5]. In the present case, it remains uncertain whether hypoxic ischemia alone, or the combination with benzodiazepine intoxication, is responsible for selective involvement of the basal ganglia.

We conclude that although rare, selective delayed anoxic injury of the basal ganglia may cause an isolated subacute hypokinetic-rigid syndrome in adulthood. The lucid interval after a circulatory arrest, the symmetrical appearance of the hypokinetic-rigid syndrome and the hyperintense signal of the striatum on T2-MRI contributed to the diagnosis.
